# Effect of reduction quality on post-operative outcomes in 31-A2 intertrochanteric fractures following intramedullary fixation: a retrospective study based on computerised tomography  findings

**DOI:** 10.1007/s00264-018-4098-1

**Published:** 2018-08-16

**Authors:** Jiantao Li, Licheng Zhang, Hao Zhang, Peng Yin, Mingxing Lei, Guoqi Wang, Song Wang, Peifu Tang

**Affiliations:** 1 Department of Orthopaedics, Chinese PLA General Hospital, No. 28 Fuxing Road, Beijing, 100853 China; 2 Department of Orthopedics, Beijing Chaoyang Hospital, Capital Medical University, No. 8 Gong Ren Ti Yu Chang Nan Lu Rd, Beijing, 100020 China; 3 Department of Orthopaedics, Hainan Branch of Chinese PLA General Hospital, Sanya, 572013 Hainan China; 4 Medical College, Nankai University, No. 94 Weijin Road, Tianjin, 300071 China

**Keywords:** Intertrochanteric fractures, Intramedullary nail, Reduction quality, Outcomes, CT findings

## Abstract

**Purpose:**

To determine how the reduction of medial and anteromedial cortices using CT findings in 31-A2 intertrochanteric fractures treated with the intramedullary nail could affect the clinical outcomes and complication rates of the fractures.

**Methods:**

We retrospectively analyzed the data of 43 patients with 31-A2 intertrochanteric fractures who underwent closed reduction and intramedullary internal fixation (CRIF) between January 2010 and December 2013. Patients were classified into two groups based on the post-operative CT scans taken from the sagittal and coronal planes, respectively. Five radiographic parameters and three clinical parameters were used to evaluate the post-operative functional states and mobilization levels in this study. Post-operative complications were also recorded.

**Results:**

The mean loss of the femoral neck-shaft angle (FNSA) was significantly smaller in Group C1 than that in Group C2. There were significant differences in the sliding distance of the cephalic nail and the loss of femoral head height between the two groups. In terms of the reduction conditions shown on the sagittal planes, the FNSA, sliding distance of the cephalic nail, and the loss of FHH were significantly different, although differences in TCD were not significant. Patients in groups C1 (3.6%) and S1 (0.0%) had lower complication rates compared to patients in groups C2 (26.7%) and S2 (27.8%).

**Conclusion:**

Patients with good reduction quality of the medial and anteromedial sustainable cortices had better clinical outcomes and lower complication rates. The sustainable stability and anti-rotational function of these validated reductions might play a critical role in maintaining the fragment positions and reducing the incidence of complications in patients.

**Electronic supplementary material:**

The online version of this article (10.1007/s00264-018-4098-1) contains supplementary material, which is available to authorized users.

## Introduction

Intertrochanteric fractures are the most common fractures especially among elderly patients, which are serious medical, social, economic healthcare problem [1] and often lead to a substantial decline in function and independence [2]. Early surgical intervention is the optimal strategy that can enable early mobilization [3], and also provide humanitarian relief for patients [4]. Intramedullary fixation has been applied in more cases in recent years due to its theoretic advantages and biomechanical superiority over extramedullary fixation, especially in the unstable fracture patterns [5]. However, complication rates of up to 20.5% have been reported using the intramedullary nail technique [6]. Among all the complications, the two most common ones are excessive sliding of the cephalic nail [6] and cutout [7].

Kaufer et al. [8] reported five major factors associated with these complications: bone quality, fragment patterns, implant type, placement of implant, and quality of reduction. Haidukewych et al. [9] also pointed out that the first two factors cannot be controlled, but the morbidity associated with the fractures could be minimized by selecting the appropriate implants, inserting them in the ideal positions, as well as performing the validated reduction. The concept of tip-apex distance (TAD) can be used to guide the nail into proper placement to avoid cutout [10]. Other efforts have been made to demonstrate that reduction of the medial cortex shown on X-ray radiographs relate to less mechanical complication rates [6, 11–15]. Moreover, plain radiographs tend to be less accurate and cannot elaborately display the exact reduction area of the three-dimensional intertrochanteric structures. Even though Lechler et al. [16] and Bücking et al. [17] developed a method of assessing parameters of the proximal femur using anteroposterior radiographs to avoid projection errors, a high variability of hip rotation was found on the plain radiographs. The best radiological means of measuring femoral parameters is CT [16].

To the best of our knowledge, no clinical study has been performed to investigate the reduction quality particularly of 31-A2 fractures using CT findings to overcome the limitations of X-ray radiographs. Unstable fracture types of the AO/OTA 31-A2 classification are characterized by the posterior cortex of the intertrochanteric crest being discontinued or comminuted with lesser trochanteric detachment [18]. The reduction of the medial or anteromedial cortices should provide necessary impaction when fractures are fixed with the intramedullary nail. Therefore, the purpose of the current study is to determine how the reduction of medial and anteromedial cortices using CT findings in 31-A2 intertrochanteric fractures treated with the intramedullary nail could affect the clinical outcomes and complication rates of the fractures.

## Materials and methods

### Patient selection

**Fig. 1:**
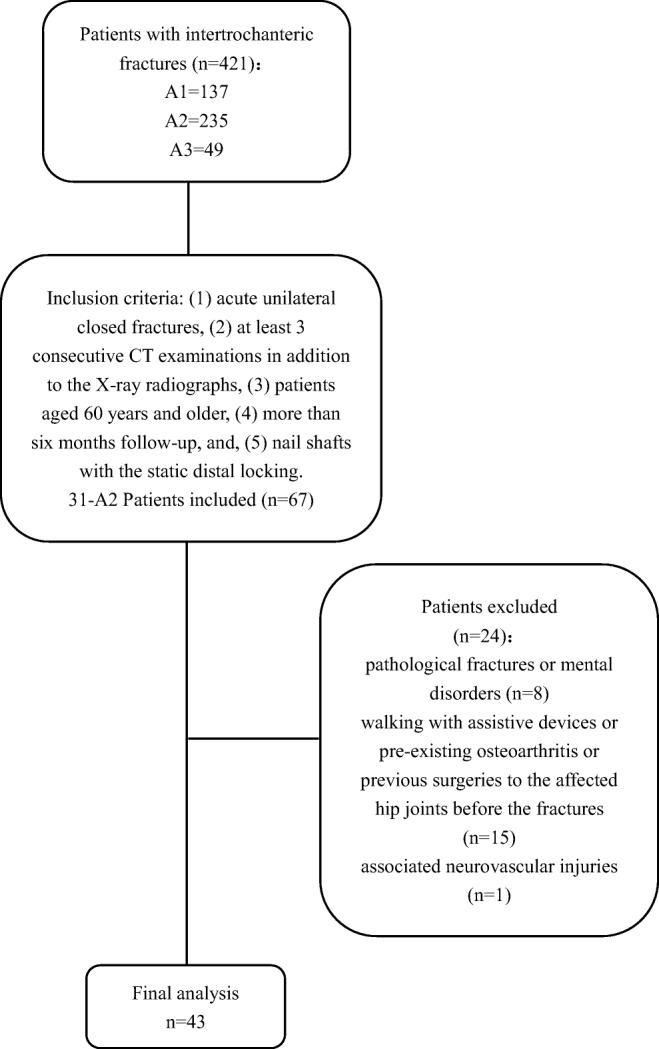
Flow chart for inclusion and exclusion of patients

The study comprised 43 patients with 31-A2 intertrochanteric fractures who underwent closed reduction and internal fixation (CRIF) with the intramedullary nail at our institution between January 2010 and December 2013. A flow chart of the inclusion and exclusion process was given in Fig. 1.

This study was approved by the Ethics Committee of the Chinese PLA General Hospital. A written informed consent was obtained from all the patients.

Pre-operative radiographs and CT scans were evaluated by a senior orthopaedic surgeon to determine the AO/OTA fracture types. All the surgery was performed by senior orthopaedic surgeons. All interlocked femoral nails were locked distally in the static position. In this study, all patients were treated with short nails.

All the patients participated in similar rehabilitation protocols. Patients on the second day post-operatively were mobilized with unrestricted weight-bearing. Post-operative radiographs and CT scans were also performed to evaluate the reduction and fixation positions of the patients.

### Creation of new coronal planes

**Fig. 2:**
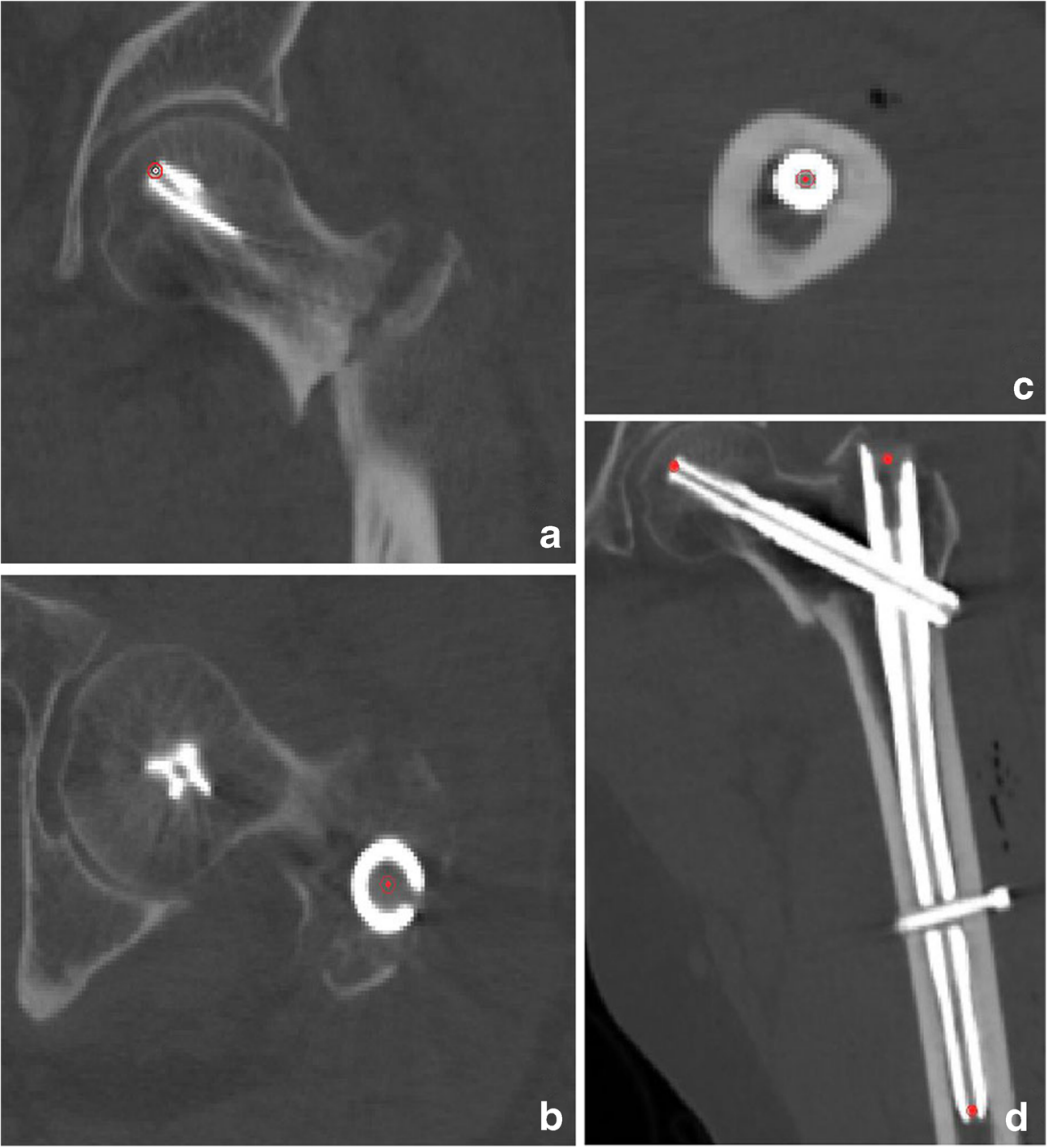
**a** The first point. **b** The second point. **c** The third point. **d** New coronal planes through three points were created

DICOM data were collected from each patient and imported into the Mimics 16.0 (Materialise, Leuven, Belgium). With the use of resliced functions, new coronal planes through three points were created: The first point was located on the tip of the cephalic nail; the second point was on the proximal circle centre of the shaft nail; the third point was on the most remote circular centre of the shaft nail. Through these three points, new coronal planes based on the intramedullary nail were created (Fig. 2). New transverse and sagittal planes based on the new coronal plane were also automatically created. The new orthogonal images could be visualized layer-by-layer using Mimics.

### Patient classification

**Fig. 3:**
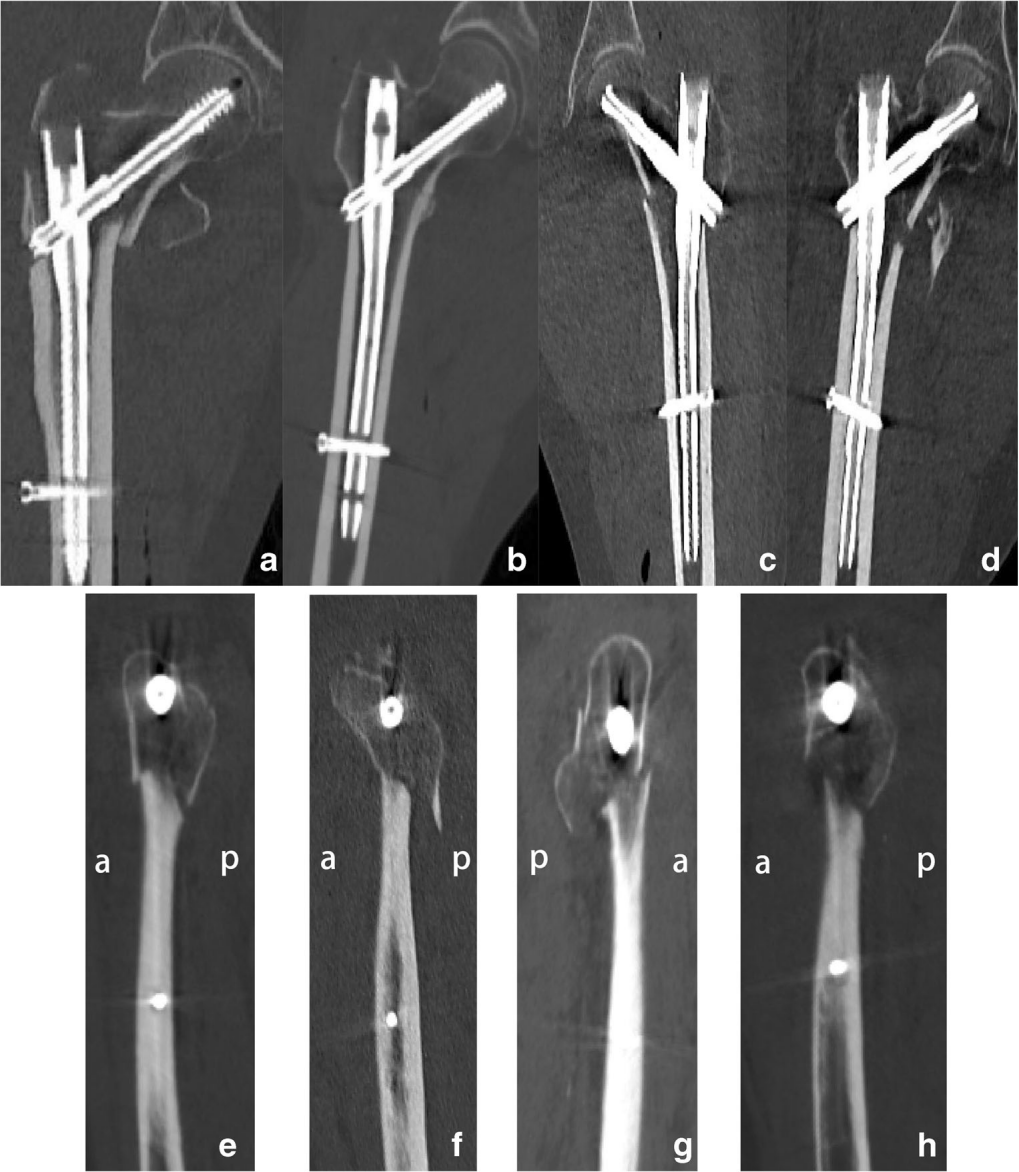
**a** The femoral neck was reduced medially to the distal fragment. **b** Medial cortices were integrated to each other. **c** The femoral neck was reduced laterally to the distal fragment. **d** Large gaps was wider than 4 mm within the medial cortices. **e** The anteromedial cortices of proximal fragment were anteriorly reduced. **f** The cortices were without displacement. **g** The anteromedial cortices were posteriorly reduced to the distal fragment. **h** Large gaps within the anteromedial cortices were wider than 4 mm

The newly resliced coronal planes were used to evaluate the quality of the fracture reduction. The patients were classified into two groups based on the CT findings of the medial cortical condition performed within one week post-operatively. Patients with the femoral neck reduced medially to the distal fragment or the medial cortices integrated to each other belonged to group C1. Patients with the femoral neck reduced laterally to the distal fragment or with large gaps wider than 4 mm [10] within the medial cortices belonged to group C2 (Fig. 3**a**–**d**). Patients were also classified into two groups based on the anteromedial cortical conditions as indicated on the new sagittal planes. Patients without displacements or the anteromedial cortices of the proximal fragments anteriorly reduced were classified as group S1. Patients with the anteromedial cortices of proximal fragments posteriorly reduced to the distal fragment or gaps within the anteromedial cortices larger than 4 mm [10] were classified as group S2 (Fig. 3**e**–h). Combined reduction types were also recorded.

### Radiographic parameters

**Fig. 4:**
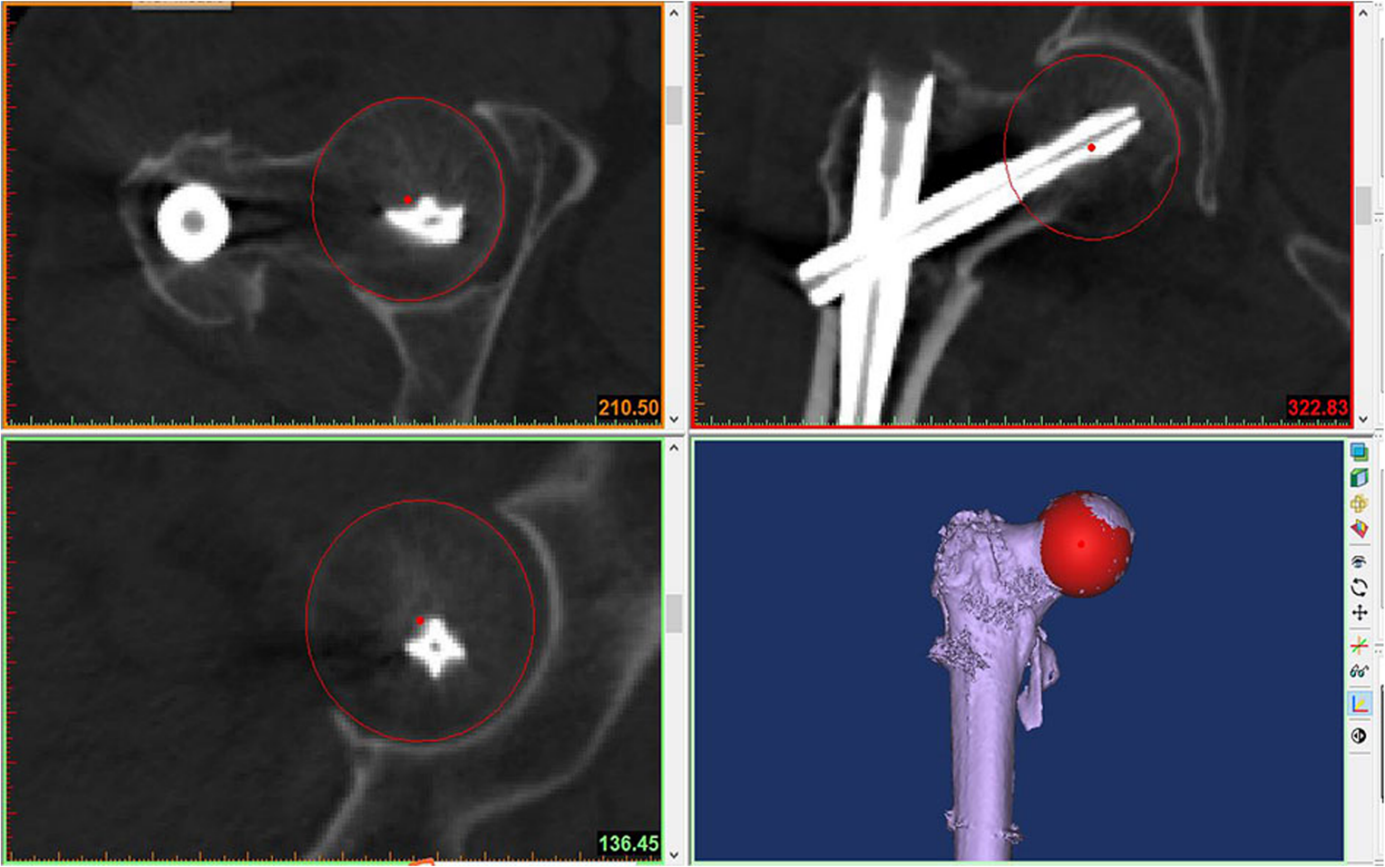
The constructed sphere fitted the contour of the femoral head properly and the cross-section of the sphere could be visualized on images in the three views

**Fig. 5:**
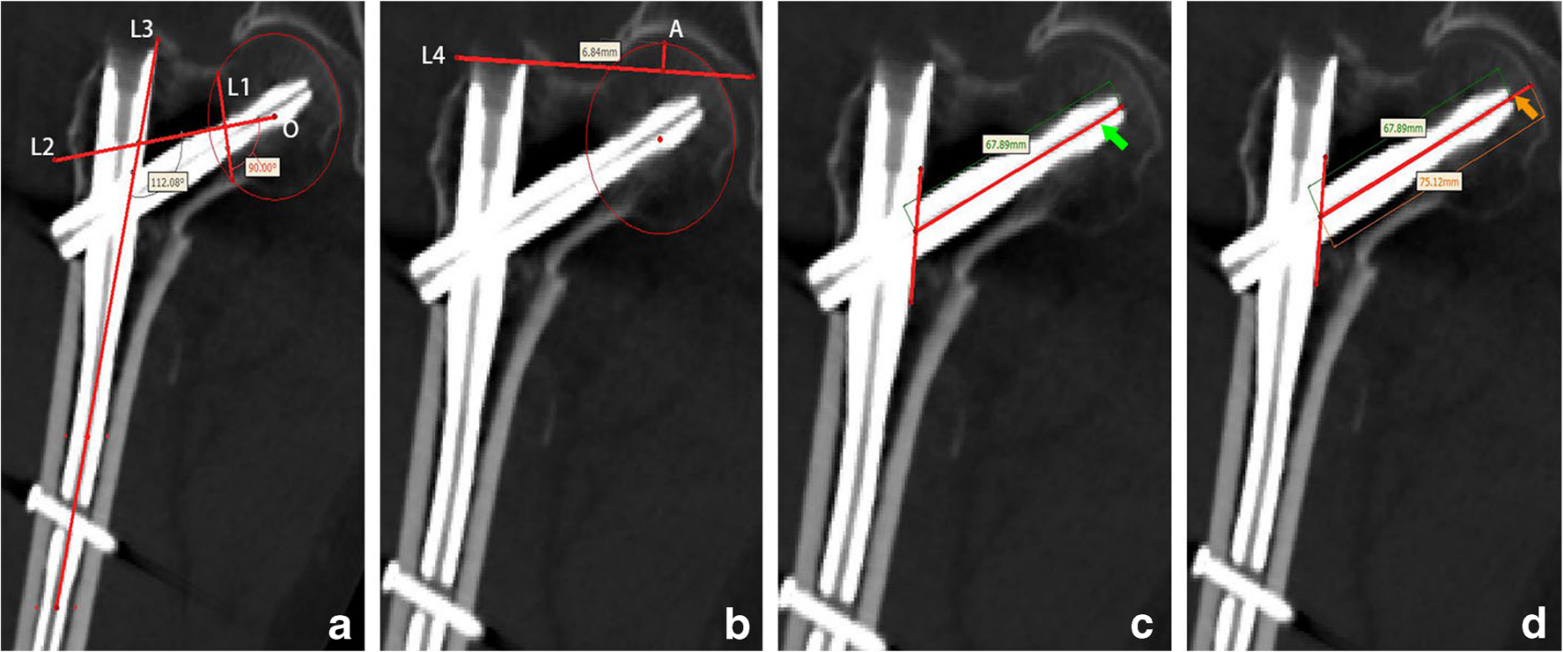
**a** L1 was the chord of the circle O and L2 was treated as the axis of the femoral neck. L3 was the axis of the femoral shaft. The angle formed between L2 and L3 was FNSA. **b** The distance between the point A and L4 was measured and designated as the FHH. **c** The green arrow marked was the *L*_mcn_. **d***L*_mtc_ (marked by the orange arrow) was overlapping *L*_mcn_ and extending to the femoral head cortex. The difference between *L*_mtc_ and *L*_mcn_ was TCD

For this study, the immediate post-operative CT findings and the 3-month follow-up data were both used for measurements. All the measurements were performed by an independent blinded observer. Parameters measured on the newly resliced coronal plane have been described as follows:

Through the newly resliced orthogonal planes, a sphere was created to simulate the femoral head. We chose the three planes that showed the best curve of the femoral head. We marked four points on the cortex of the femoral head using the MedCAD module function. A sphere approximately matching the femoral head was created through the four points. During the marking, we confirmed that all the points were distributed in different planes. There were two points existing in one plane, distant from each other. The centre of the sphere was marked point O, and the cross-section of the sphere could be visualized on images in the three views (Fig. 4). On the coronal plane, we labeled the plane where the intramedullary nail formed the largest hollow morphology as P1. We drew line L1 as the chord of the circle on P1 using two intersections generated by the circle, crossing the upper and lower cortices of the femoral neck. We then created line L2 from O perpendicular to L1. L2 was treated as the axis of the femoral neck (Fig. 5a).

On the new axial view, we chose two planes, each of which was 2 cm apart from the remote locking screw proximally and remotely. We drew circles that approximately fitted into the medullary spaces on these two planes. On P1, we created the axial line (L3) of the femoral shaft from the intersections with the circle centres.

Parameters were calculated as follows:

1. Femoral neck-shaft angle (FNSA)

Using the Mimics measurement function, we measured the angle formed between L2 and L3 on P1, designated as FNSA (Fig. 5a).

2. Femoral head height (FHH)

The FHH relative to the nail was measured, allowing for the subsequent loss of reduction analysis. This measurement was done on P1. First, we drew line L4 at the top edge of the shaft nail. We then selected point A at the superior edge of the femoral head, and the distance between point A and L4 was measured and designated as the FHH (Fig. 5b).

3. The length of medial cephalic nail (*L*_mcn_)

*L*_mcn_ was the distance between the tip of the cephalic nail and medial side of the shaft nail. The sliding distance of the cephalic nail was calculated by the *L*_mcn_ parameter in two periods (Fig. 5c).

4. Tip-cortex distance (TCD)

TCD was the distance from the tip of cephalic nail to the femoral head cortex, and it was calculated by two length parameters namely, *L*_mcn_ and *L*_mtc_. *L*_mtc_ was the line overlapping *L*_mcn_ in extension to the femoral head cortex. The difference between *L*_mtc_ and *L*_mcn_ was TCD (Fig. 5d).

5. Tip-apex distance (TAD)

We calculated TAD as described by Baumgaertner et al. [10] using the anteroposterior and lateral radiographs to evaluate the implant position.

The immediate post-operative and three month follow-up parameters of FNSA, FHH, 004C_mcn_, and TCD respectively were documented. The changes between them were calculated by subtracting the three month follow-up parameters from the immediate post-operative parameters.

### Clinical parameters

At the final follow-up, Harris hip score (HHS) [19], timed “Up & Go” (TUG) test (measuring the time taken to rise from a sitting position and walk for 20 m) [20], and the Parker-Palmer mobility score [21] were performed to evaluate the functional states and mobility levels.

### Complications

Loss of reduction, cutout, excessive sliding of the cephalic nail, and implant breakage were some of the complications recorded during the follow-up. We defined loss of reduction as the loss of NSA greater than 10°. Excessive sliding was defined as sliding distance ≥ 10 mm.

### Statistical analysis

Data analyses were performed using SPSS (version 19.0, SPSS Inc., Chicago, IL, USA). We used the chi-square and Fisher’s exact tests, respectively to analyze the categorical variables between the groups in terms of sex, fracture classification, and fixation type. Student’s *t* test was used to compare the continuous variables between the groups. Continued adjusted chi-square test was used to compare the complication data between the groups. The level of significance was set at *P* < 0.05.

#### Availability of data and materials

Data and materials relating to the study were accessed from the case systems at our department.

## Results

The patients we recruited include 17 males and 26 females with the mean age of 76.5 years at the time of the surgery (range 60–94 years). Based on the AO/OTA classification, 14 cases were classified as 31-A2.1 type, 16 as 31-A2.2 type, and 13 as 31-A2.3 type. The mean follow-up period was 13 months (range 6–23 months). Details of all cases were seen in supplement 1.

According to the reduction conditions showed on the newly resliced coronal and sagittal planes, there were 28 cases (65%) in group C1 and 15 (35%) in group C2, 25 cases (58%) in group S1 and 18 (42%) in group S2. There were four combined reduction groups: C1S1 (24 cases, 56%), C1S2 (4 cases, 9%), C2S1 (1 case, 2%), and C2S2 (14 cases, 33%). Detailed patient characteristics with all individual measurements were presented in supplement 2. C1S1 was classified into group 1 (both reconstruction), and C1S2, C2S1, C2S2 were classified into group 2 (not both reconstruction). The radiographic parameters were shown in Table 1.

**Table 1:** Patients demographics and operative data based on the group respecting coronal, sagittal, and combined reconstruction planes

Description		Group C1	Group C2	*P* value	Group S1	Group S2	*P* value	Group 1 (both reconstruction)	Group 2 (not both reconstruction)	*P* value
Total number		28	15		25	18		24	19	
Gender	Females	17	9	0.964	16	10	0.808	15	11	0.759
	Males	11	6		9	8		9	8	
Mean age (year)		75.7	77.9	0.474	75.4	78.0	0.363	75.5	77.7	0.449
AO classification	31-A2.1	7	7	0.179	8	6	0.058	7	7	0.124
	31-A2.2	13	3		12	4		12	4	
	31-A2.3	8	5		4	9		5	8	
Fixation type	Gamma 3	7	1	0.264	6	2	0.462	6	2	0.462
	Intertan	2	2		2	2		2	2	
	PFNA	19	12		16	15		16	15	
TAD (mm)		20.7	18.9	0.331	20.9	18.8	0.245	21.0	18.8	0.228
Change of FNSA (°)		− 0.88	− 3.51	0.016*	− 0.73	− 3.27	0.016*	− 0.68	− 3.21	0.016*
Sliding distance of cephalic nail (mm)		− 0.47	− 3.47	0.006*	− 0.39	− 3.09	0.004*	− 0.35	− 3.00	0.003*
Change of TCD (mm)		− 0.33	− 0.01	0.528	− 0.39	0.02	0.433	− 0.42	0.04	0.364
Change of FHH (mm)		− 0.99	− 2.77	0.026*	− 0.95	− 2.53	0.041*	− 1.00	− 2.39	0.072

Abbreviation: *TAD*, tip-apex distance; *FNSA*, femoral neck-shaft angle; *TCD*, tip-cortex distance; *FHH*, femoral head height

* *P* < 0.05

At the final follow-up, the mean HHS and Parker-Palmer mobility scores were significantly different between groups, and mean TUG test time was similar between groups (Table 2).

**Table 2:** Functional data at the latest follow-up in the different reduction groups

Clinical parameters	Coronal group		*P* value	Sagittal group		*P* value	Combined group		*P* value
	C1	C2		S1	S2		Group 1 (both reconstruction)	Group 2 (not both reconstruction)	
HHS (points)	77 (67–87)	73 (67–82)	0.011*	78 (67–87)	74 (67–84)	0.021*	78 (67–87)	74 (67–84)	0.017*
TUG (sec)	24 (19–35)	26 (23–36)	0.114	24 (19–35)	26 (20–36)	0.164	24 (19–35)	26 (20–36)	0.214
P-P score (points)	6.9 (4–9)	6.5 (5–8)	0.227	7.0 (4–9)	6.3 (5–8)	0.035*	7.0 (4–9)	6.4 (5–8)	0.043*

Abbreviation: *HHS*, Harris hip score; *TUG*, timed “Up & Go”; *P-P score*, Parker and Palmer score

* *P* < 0.05

Five patients (11.6%) experienced fracture-related complications. Further details were listed in Table 3.

**Table 3:** Mechanical failures of patients in different reduction groups. *P* was obtained from continued adjusted chi-square test

Complications	Group C		*P* value	Group S		*P* value	Combined group				*P* value
	C1	C2		S1	S2		C1S1	C1S2	C2S1	C2S2	
Yes	1^a^	4^b^	0.043	0	5^c^	< 0.01	0	1^d^	0	4^e^	0.022
No	27	11		25	13		24	3	1	10	
Total	28	15		25	18		24	4	1	14	

^a^Loss of reduction, 1 case

^b^Loss of reduction, 2 cases; excessive sliding of cephalic nail, 2 cases

^c^Loss of reduction, 3 cases; excessive sliding of cephalic nail, 2 cases

^d^Loss of reduction, 1 case

^e^Loss of reduction, 2 cases; excessive sliding of cephalic nail, 2 cases

## Discussion

In this retrospective clinical study, we determined how anterior and anteromedial cortices reduction condition contributed to the clinical outcome of intramedullary fixation-treated 31-A2 intertrochanteric fractures. To test this hypothesis, we adopted an approach that showed how the reduction types and quality affects the anatomic parameters, clinical outcomes, and post-operative functions. We found that patients with high-quality reduction experienced better clinical outcomes and lower complication rates compared to those without the reconstruction. Our study showed that reconstruction of medial and anteromedial cortices played an important role in the treatment of unstable intertrochanteric fractures.

The medial and anteromedial reduction conditions on the newly resliced planes were elaborately elucidated in this study. Patients without medial cortex reconstruction (group C2), although fixed with the intramedullary nail, failed to keep the position of the fracture fragments stable, which was consistent with previous reports [15]. In addition, patients in group S2 were less stable compared to those in group S1, which is in agreement with other studies [12–14]. The combined reconstruction type also indicates the necessity of the medial and anteromedial cortex reconstruction. However, in this study, with the reconstruction of the newly resliced planes and the measurement of all data using the Mimics software, we obtained precise values and accurately pointed out the critical areas of the reduction (as shown in Fig. 3), which provides significant insights on the treatment of the intertrochanteric fractures.

A previous study showed that the complication rate of the intertrochanteric fractures treated with the intramedullary implants was up to 20.5% [6], and their studies suggested that patients with stable fracture pattern and good reduction were less likely to suffer mechanical complications. Wu et al. [22] concluded that the fixation devices used in unstable fractures were more likely to fail. Therefore, in this unstable intertrochanteric fracture study, good reduction played a key role in reducing the complications. The five patients who underwent unsatisfactory reduction of the medial or anteromedial cortices experienced fracture-related complications. Conversely, patients who underwent reduction at both sites did not have any complications (Table 3).

The 31-A2 fracture type classification was characterized by the posterior cortex of the intertrochanteric crest being discontinued or comminuted with lesser trochanteric detachment [18]. Even though individual experience is fundamental for a majority of act of osteosynthesis [23], surgeons cannot reduce the abovementioned bony structures despite the concerns raised during the closed reduction and internal fixation operation using the intramedullary nail. The fundamental purpose of intertrochanteric fracture treatment is to effectively restore pre-operative functional and motor states [24]. Therefore, the reconstruction of medial and anteromedial cortices has become necessary. In our clinical experience, the major effects of medial and anteromedial cortices are as follows: (1) the medial cortex can provide sustainable stability and prevent the femoral head varus as well as proximal fragments from sliding laterally; (2) the anteromedial cortex can retain the anteversion angle; (3) both reduction sites of the cortices present in different planes can prevent the rotation of the proximal femoral components; (4) the abovementioned functions act as keystones providing rigid buttress among the fragments to prevent complications like cutout, varus angulation, excessive telescoping, and re-operation. When the reconstruction of the sustainable cortices fails, the proximal fragments tend towards lateralization or rotation without the posteromedial lesser trochanter or posterolateral intertrochanteric crest block, which leads to the collapse or the regain of another stability style. This regained stability (we renamed it as secondary stability) could be implemented through the impaction of the fragments or the buttress from the nail inserted in the proper position but generally with lower physical functional scores. Currently, the device withstands most of the forces associated with daily activities, which could be at risk for hardware failure. The earlier the secondary stability is feasible, the less hardware failure occurs.

To the best of our knowledge, this is the first study to investigate the effects of reduction quality on the outcomes after intramedullary fixation of 31-A2 intertrochanteric fractures by assessing the reconstruction of the medial and anteromedial cortices using CT findings. We introduced the concept of sustainable cortices and demonstrated their essential functions in clinical practice when used to treat unstable intertrochanteric fractures. However, our study was retrospective in nature and the sample size was relatively small. Therefore, multi-centered, prospective, randomized-control trials are recommended to overcome the limitations of this study in the future.

In conclusion, the results of this study showed that patients with high reduction quality of medial and anteromedial sustainable cortices had better clinical outcomes and lower complication rates. The sustainable stability and anti-rotational function of these validated reductions might play a critical role in maintaining the fragment positions and reducing the incidence of complications in patients.

## Electronic supplementary material

ESM 1 (DOCX 15 kb)

ESM 2 (XLSX 20 kb)

## Abbreviations

TAD: tip-apex distance
FNSA: femoral neck-shaft angle
TCD: tip-cortex distance
FHH: femoral head height

## References

[1] Lizaur-Utrilla A, Gonzalez-Navarro B, Vizcaya-Moreno MF, Miralles Munoz FA, Gonzalez-Parreno S, Lopez-Prats FA (2018) Reasons for delaying surgery following hip fractures and its impact on one year mortality. Int Orthop. 10.1007/s00264-018-3936-5 10.1007/s00264-018-3936-5 29744645

[2] Stone AV, Jinnah A, Wells BJ, Atkinson H, Miller AN, Futrell WM, Lenoir K, Emory CL (2018). Nutritional markers may identify patients with greater risk of re-admission after geriatric hip fractures. Int Orthop.

[3] Weller I, Wai E, Jaglal S, Kreder H (2005). The effect of hospital type and surgical delay on mortality after surgery for hip fracture. The Journal of Bone and Joint Surgery British Volume.

[4] Wong SHJ, Fang XC, Yee KHD, Wong TM, Pun CTT, Lau TW, Leung KLF (2018). Hip fracture time-to-surgery and mortality revisited: mitigating comorbidity confounding by effect of holidays on surgical timing. Int Orthop.

[5] Anglen JO, Weinstein JN (2008). Nail or plate fixation of intertrochanteric hip fractures: changing pattern of practice: a review of the American Board of Orthopaedic Surgery Database. J Bone Joint Surg Am.

[6] Liu W, Zhou D, Liu F, Weaver MJ, Vrahas MS (2013). Mechanical complications of intertrochanteric hip fractures treated with trochanteric femoral nails. J Trauma Acute Care Surg.

[7] Lobo-Escolar A, Joven E, Iglesias D, Herrera A (2010). Predictive factors for cutting-out in femoral intramedullary nailing. Injury.

[8] Kaufer H (1980). Mechanics of the treatment of hip injuries. Clin Orthop Relat Res.

[9] Haidukewych G (2009). Intertrochanteric fractures: ten tips to improve results. J Bone Joint Surg Am.

[10] Baumgaertner MR, Curtin SL, Lindskog DM, Keggi JM (1995). The value of the tip-apex distance in predicting failure of fixation of peritrochanteric fractures of the hip. J Bone Joint Surg Am.

[11] Li C, Xie B, Chen S, Lin G, Yang G, Zhang L (2016). The effect of local bone density on mechanical failure after internal fixation of pertrochanteric fractures. Arch Orthop Trauma Surg.

[12] Ito J, Takakubo Y, Sasaki K, Sasaki J, Owashi K, Takagi M (2015). Prevention of excessive postoperative sliding of the short femoral nail in femoral trochanteric fractures. Arch Orthop Trauma Surg.

[13] Kozono N, Ikemura S, Yamashita A, Harada T, Watanabe T, Shirasawa K (2014). Direct reduction may need to be considered to avoid postoperative subtype P in patients with an unstable trochanteric fracture: a retrospective study using a multivariate analysis. Arch Orthop Trauma Surg.

[14] Tsukada S, Okumura G, Matsueda M (2012). Postoperative stability on lateral radiographs in the surgical treatment of pertrochanteric hip fractures. Arch Orthop Trauma Surg.

[15] Chang SM, Zhang YQ, Ma Z, Li Q, Dargel J, Eysel P (2015). Fracture reduction with positive medial cortical support: a key element in stability reconstruction for the unstable pertrochanteric hip fractures. Arch Orthop Trauma Surg.

[16] Lechler P, Frink M, Gulati A, Murray D, Renkawitz T, Bucking B, Ruchholtz S, Boese CK (2014). The influence of hip rotation on femoral offset in plain radiographs. Acta Orthop.

[17] Buecking B, Boese C, Seifert V, Ruchholtz S, Frink M, Lechler P (2015). Femoral offset following trochanteric femoral fractures: a prospective observational study. Injury.

[18] Müller ME, Koch P, Nazarian S, Schatzker J (1990). The comprehensive classification of fractures of long bones.

[19] Harris WH (1969). Traumatic arthritis of the hip after dislocation and acetabular fractures: treatment by mold arthroplasty. An end-result study using a new method of result evaluation. J Bone Joint Surg Am.

[20] Reindl R, Harvey E, Berry G, Rahme E (2015). Intramedullary versus extramedullary fixation for unstable intertrochanteric fractures: a prospective randomized controlled trial. J Bone Joint Surg Am.

[21] Parker M, Palmer C (1993). A new mobility score for predicting mortality after hip fracture. The Journal of Bone and Joint Surgery British Volume.

[22] Wu CC, Tai CL (2010). Effect of lag-screw positions on modes of fixation failure in elderly patients with unstable intertrochanteric fractures of the femur. J Orthop Surg.

[23] Papin P, Berthonnaud E (2017). Incidence of osteosynthesis of members in France. Int Orthop.

[24] Moyet J, Deschasse G, Marquant B, Mertl P, Bloch F (2018) Which is the optimal orthogeriatric care model to prevent mortality of elderly subjects post hip fractures? A systematic review and meta-analysis based on current clinical practice. Int Orthop. 10.1007/s00264-018-3928-5 10.1007/s00264-018-3928-5 29691612

