# Supplementary material for: Effect of reduction quality on post-operative outcomes in 31-A2 intertrochanteric fractures following intramedullary fixation: a retrospective study based on computerised tomography findings
Source: Int Orthop. 2018 Aug 16;43(8):1951–9. doi: 10.1007/s00264-018-4098-1 (PMC6647079; doi:10.1007/s00264-018-4098-1)
Supplement: Supplementary file 1 — (DOCX 15 kb) [file 264_2018_4098_MOESM1_ESM.docx]

| **Table 1** Cross table of groups respecting both reconstruction planes (sagittal and coronal). | | | | |
| --- | --- | --- | --- | --- |
|  |  | Group S | | No. of cases |
|  |  | S1 | S2 |  |
| Group C | C1 | 24 | 4 | 28 |
|  | C2 | 1 | 14 | 15 |
| No. of cases |  | 25 | 18 | Total: 43 |

| **Table 2** Patients outcome data based on the implant type. | | | | | |
| --- | --- | --- | --- | --- | --- |
| Description |  | PFNA | InterTan | Gamma 3 | P value |
| Total number |  | 31 | 4 | 8 |  |
| AO classification | 31-A2.1 | 11 | 2 | 1 | 0.276 |
|  | 31-A2.2 | 9 | 2 | 5 |  |
|  | 31-A2.3 | 11 | 0 | 2 |  |
| TAD (mm) |  | 20.1 | 18.5 | 20.5 | 0.845 |
| Change of FNSA (°) |  | -1.68 | -0.21 | -3.03 | 0.400 |
| Sliding distance of cephalic nail (mm) |  | -2.01 | -0.21 | -0.27 | 0.165 |
| Change of TCD (mm) |  | -0.08 | -0.37 | -0.67 | 0.660 |
| Change of FHH (mm) |  | -1.77 | 0.23 | -1.91 | 0.315 |
| HHS (points) |  | 75 | 79 | 77 | 0.442 |
| TUG (sec) |  | 25 | 23 | 24 | 0.933 |
| P-P score (points) |  | 6.7 | 6.8 | 6.9 | 0.610 |
| Complication (numbers) |  | 4 | 0 | 1 | 0.748 |
